# Systems-Level Immunomonitoring from Acute to Recovery Phase of Severe COVID-19

**DOI:** 10.1016/j.xcrm.2020.100078

**Published:** 2020-08-05

**Authors:** Lucie Rodriguez, Pirkka T. Pekkarinen, Tadepally Lakshmikanth, Ziyang Tan, Camila Rosat Consiglio, Christian Pou, Yang Chen, Constantin Habimana Mugabo, Ngoc Anh Nguyen, Kirsten Nowlan, Tomas Strandin, Lev Levanov, Jaromir Mikes, Jun Wang, Anu Kantele, Jussi Hepojoki, Olli Vapalahti, Santtu Heinonen, Eliisa Kekäläinen, Petter Brodin

**Affiliations:** 1 Science for Life Laboratory, Department of Women’s and Children’s Health, Karolinska Institutet, Solna 171 77, Sweden; 2 Division of Intensive Care Medicine, Department of Anesthesiology, Intensive Care and Pain Medicine, University of Helsinki, and Helsinki University Hospital, Helsinki 00100, Finland; 3 Translational Immunology Research Program, University of Helsinki, and Helsinki University Hospital, Helsinki 00100, Finland; 4 Department of Virology, University of Helsinki, and Helsinki University Hospital, Helsinki 00100, Finland; 5 Inflammation Center, Division of Infectious Diseases, University of Helsinki, and Helsinki University Hospital, Helsinki 00100, Finland; 6 New Children’s Hospital, Pediatric Research Center, University of Helsinki, and Helsinki University Hospital, Helsinki 00100, Finland; 7 Department of Pediatric Rheumatology, Karolinska University Hospital, Solna 171 76, Sweden

**Keywords:** human immunology, systems immunology, COVID-19, SARS-CoV-2, mass cytometry, plasma proteins

## Abstract

Severe disease of SARS-CoV-2 is characterized by vigorous inflammatory responses in the lung, often with a sudden onset after 5–7 days of stable disease. Efforts to modulate this hyperinflammation and the associated acute respiratory distress syndrome rely on the unraveling of the immune cell interactions and cytokines that drive such responses. Given that every patient is captured at different stages of infection, longitudinal monitoring of the immune response is critical and systems-level analyses are required to capture cellular interactions. Here, we report on a systems-level blood immunomonitoring study of 37 adult patients diagnosed with COVID-19 and followed with up to 14 blood samples from acute to recovery phases of the disease. We describe an IFNγ-eosinophil axis activated before lung hyperinflammation and changes in cell-cell co-regulation during different stages of the disease. We also map an immune trajectory during recovery that is shared among patients with severe COVID-19.

## Highlights

Immunomonitoring from acute to recovery phase COVID-19
An IFNγ-eosinophil axis precedes lung hyperinflammation
Basophils modulate SARS-CoV-2 IgG responses
A shared trajectory of immunological recovery in COVID-19

## Graphical Abstract

**Figure:**
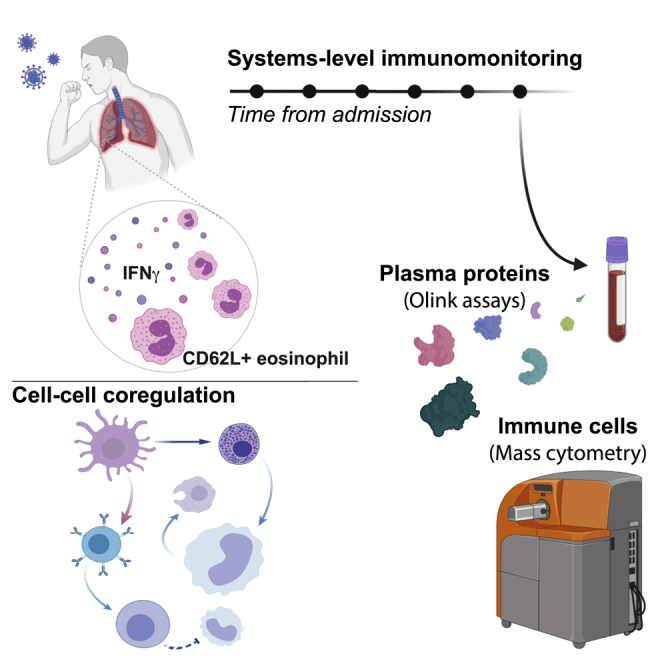

## Teaser

Immune dysregulation plays a pivotal role during severe COVID-19. Rodriguez et al. present a systems-level, longitudinal study of 37 COVID-19 patients monitored from acute to recovery phases of disease. This study reveals cell-protein dependencies and co-regulated features, as well as a shared immunological trajectory during recovery.

## Introduction

Since its emergence in December 2019, the severe acute respiratory syndrome-coronavirus 2 (SARS-CoV-2) causing coronavirus disease 2019 (COVID-19) has infected millions of individuals and caused hundreds of thousands of deaths worldwide. The betacoronavirus has a high degree of sequence homology with previous SARS-CoV and Middle East respiratory syndrome (MERS) coronaviruses and binds to the angiotensin-converting enzyme 2 (ACE2) receptor to enter cells in the respiratory and intestinal epithelium.^1^

Cells recognize the presence of the virus through pathogen-recognition receptors (PRRs) and elicit antiviral response programs.^2^ The two main components of such antiviral programs involve the production of type I and III interferons (IFNs) that induce downstream transcription of hundreds of IFN-stimulated genes (ISGs) that interfere with viral replication in the cell.^3^ The second element of the antiviral response program is the secretion of chemokines that recruit specialized cells of the immune system to clear the virus. SARS-CoV-2, like other viruses, has evolved countermeasures to these defenses, and, in particular, the virus efficiently interferes with IFN signaling and the induction of ISGs in SARS-CoV-2-infected cells.^4^^,^^5^ In contrast, pro-inflammatory cytokine and chemokine responses are induced normally, and this imbalance between antiviral and pro-inflammatory responses is a key feature of COVID-19.^6^

Another observation during the COVID-19 pandemic is the different disease courses among different individuals infected by the SARS-CoV-2 virus. Most individuals present with very mild disease, often asymptomatic, and a few develop a life-threatening disease requiring intensive care. The strongest determinant of disease severity is age, with children presenting almost exclusively with mild disease,^7^ while the elderly, those older than 70 years of age, are much more likely to develop severe COVID-19. Males and females are infected at similar rates, but males are much more likely to develop severe disease requiring intensive care.^8^ Obesity, smoking, and hypertension are other risk factors for severe COVID-19.^9^ However, COVID-19 contrasts with other respiratory viral infections in that pregnant women do not seem to be more likely to develop severe disease, and this is also true for patients with various forms of immunodeficiency. One likely reason for these observations is that severe disease is associated with exuberant immune responses and a skewed immune regulation against pro-inflammatory responses in pregnancy and T cell deficiencies in transplant patients make such hyperinflammatory responses less likely. To treat hyperinflammation in severe COVID-19, we need to better understand what cells are involved, their interactions, and protein mediators used to orchestrate their responses. To this end, we performed systems-level analyses of the immune system in blood from 37 patients, from acute to recovery phases of COVID-19, with up to 14 blood samples collected from a given patient. These analyses reveal a sequence of responses involving many immune cell populations at different stages of the disease. A transient response involving IFN-γ upregulating CD62L on eosinophils before lung hyperinflammation are exemplified when you look at coregulated cell populations, and immune correlates of productive antibody responses to SARS-CoV-2, as well as an integrated immune trajectory shared across patients recovering from severe COVID-19.

## Results

### Longitudinal Profiling of Patients with COVID-19

**Figure 1:**
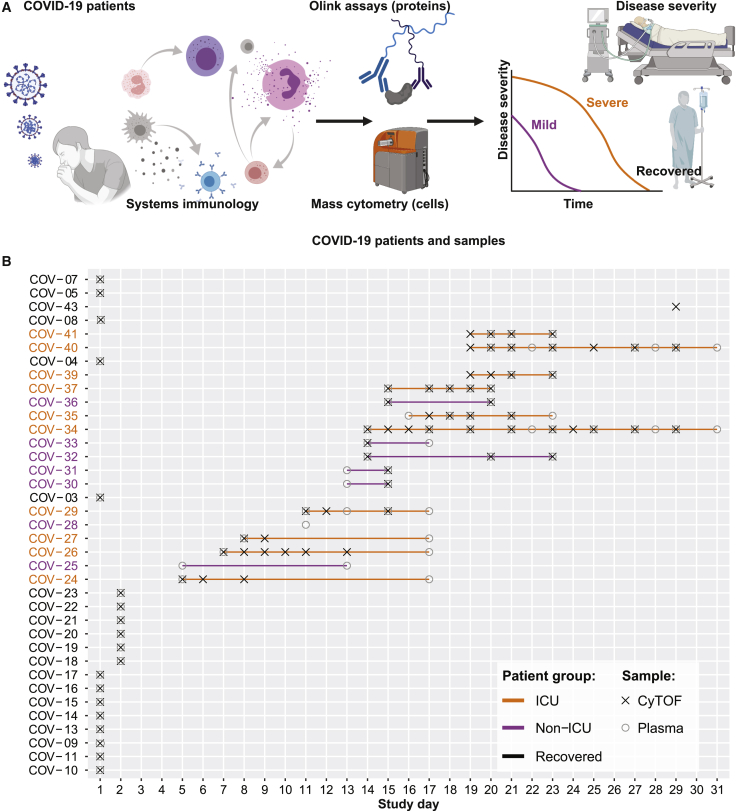
Longitudinal Profiling of the Immune System in Moderate and Severe COVID-19 (A) A total of 180 unique plasma proteins were quantified using Olink assays (n = 76 plasma samples) and whole-blood immune cells analyzed by mass cytometry (n = 78 whole-blood samples). (B) Monitoring and longitudinal sampling of blood cells (x) and plasma (o) from 37 patients at the Helsinki University Hospital, with patient groups demarcated by colored sample IDs.

Given the enormous diversity among immune systems in humans, longitudinal monitoring of patients is required to appreciate the immunological changes occurring during the disease process. Also, systems-level analyses methods such as mass cytometry^10^ enable all immune cell populations to be distinguished and analyzed in a given blood sample, allowing for coordinated changes across cell populations to be revealed. We have combined these cellular measurements with analyses of 180 unique plasma proteins using Olink analyses^11^ (Figure 1A). To understand systems-level immune responses during moderate to severe COVID-19, we monitored longitudinal samples from 17 patients, some treated in the intensive care unit (ICU) and some treated in regular hospital wards with oxygen support but no mechanical ventilation, in addition to 20 recovered patients comprised of 18 mild COVID-19 recovered patients and 2 hospitalized COVID-19 recovered patients (Figure 1B). Patients did not receive immunomodulatory therapies in this cohort apart from one before ICU admission, and the immunological changes thus reflect the natural course of severe COVID-19 infection. All of the patients in this cohort survived their infection.

### The Characteristics of Acute and Recovery Phases of COVID-19

**Figure 2:**
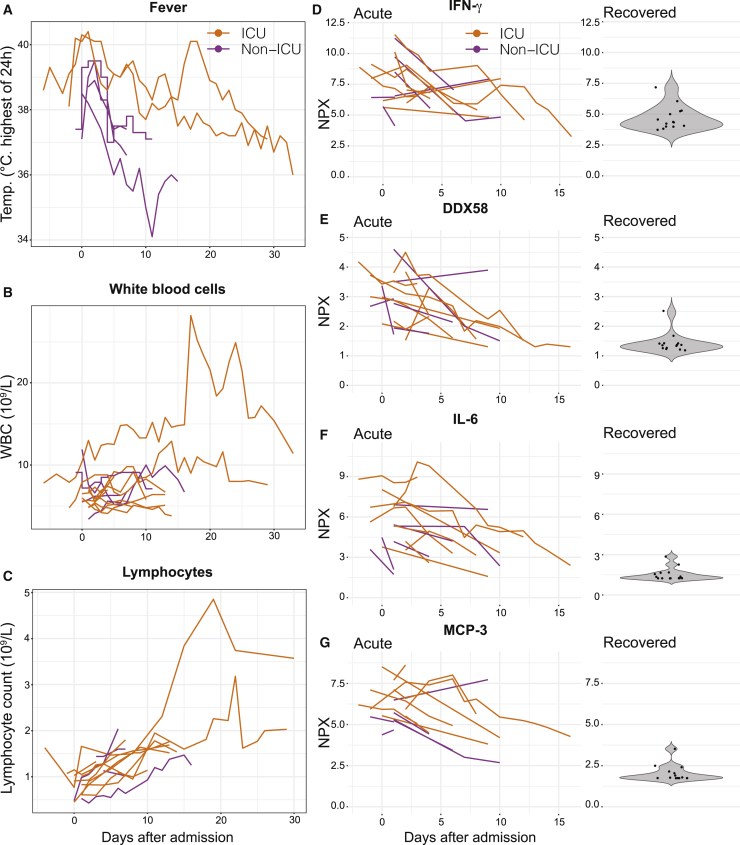
The Natural Course of Severe COVID-19 from Admission to Clinical Recovery (A) Body temperature measurements from representative patients over the course of 30 days, from admission to the hospital in ICU and non-ICU patients. (B and C) White blood cell counts (B) and lymphocyte counts (C) during acute and recovery phase in COVID-19 patients. (D–G) Plasma levels of the indicated proteins using Olink assays in longitudinal samples from 16 acute patients (left) and single measurements from 20 recovered patients (right). NPX, normalized protein expression.

Clinical measurements were taken from acute and recovered patients, including body temperature, white blood cell (WBC) counts, and lymphocyte counts. Milder cases of COVID-19 showed lower body temperatures as well as faster normalization of body temperatures compared to severe cases, which fluctuated more over time (Figure 2A). The WBC counts changed during the stages of infection. High WBC counts prototypically occur during acute inflammation and immune responses. In severe patients we observed fluctuating levels of WBC over time (Figure 2B). More important, there were no signs of secondary bacterial infection in the patients in this cohort. Lymphopenia is one of the common features of COVID-19 and the degree of lymphopenia predicts disease severity.^9^ Lymphocyte counts were measured and seemed to recover faster in milder as compared to severe cases, although this trend was not seen uniformly (Figure 2C). This is in line with other previous reports.^12^ Plasma protein levels were measured and compared among acute and recovered phases and reflect the immune dynamics of severe COVID-19 (Figures 2D–2G). Pro-inflammatory cytokines such as interleukin-6 (IL-6) and IFN-γ predict disease severity. A decreasing trend was observed in IFN-γ and IL-6 from early admission to the hospital through recovery during the weeks of the study (Figures 2D and 2F, respectively). Similarly, DDX58, the innate immune response receptor, also called RIG-I, and the monocyte chemoattractant protein MCP-3, were elevated during acute disease and decrease during recovery (Figures 2E and 2G**,** respectively).

### The Immune Cell Changes from Acute to Recovery Phases of COVID-19

**Figure 3:**
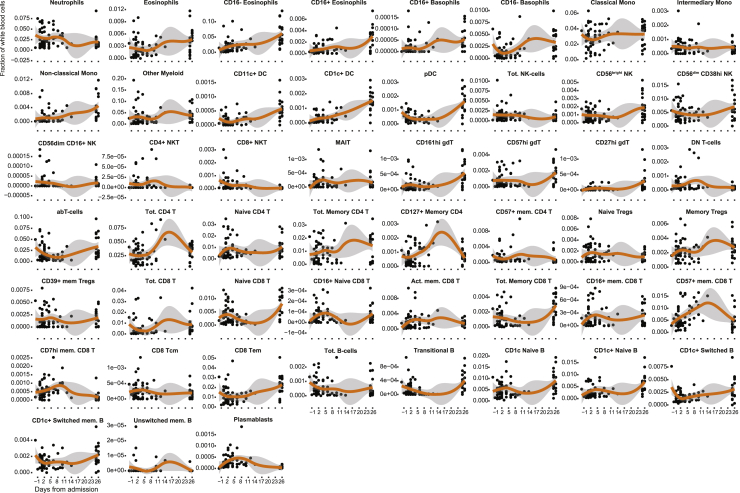
Immune Cell Proportions in COVID-19 Proportion of 57 white blood cell populations determined by mass cytometry from acute to recovery phase of COVID-19 patients (n = 35 individuals). Loess smoothing in orange. See also Figures S1 and S2.

A defining feature of the acute immune response during COVID-19 is dramatic changes in immune cell composition. These changes can be informative about likely driving factors and triggers and can help us understand the disease process better. To understand severe COVID-19 better, we plotted relative proportions of 57 immune cell populations over time in the 37 patients (Figures 3, S1, and S2). These cell populations were defined using a recently developed tool for automated cell classification based on known immune cell phenotypes.^13^ We confirmed the overrepresentation of neutrophils over lymphocytes during acute infection that is slowly reversed during the recovery phase (Figure 3). This is in line with reports suggesting that the neutrophil:lymphocyte ratio (NLR) and degree of lymphopenia are predictive of disease severity in COVID-19.^12^ The plasmablast response is clear and occurs during the first week after hospital admission in these patients (Figure 3). The recovery of T cells after the initial lymphopenia occurs over the following 2–3 weeks and is dominated by CD127-expressing effector and central memory CD4^+^ T cells, as well as CD57-expressing and differentiated memory CD8^+^ T cells (Figure 3). Also, all dendritic cell (DC) subsets increased from acute to recovery phases—CD1c^+^ DCs, CD11c^+^ DCs, and plasmacytoid DCs (pDC) (Figure 3). Despite a clear reduction in the relative abundance of neutrophils over time, the other granulocyte subsets, basophils and eosinophils, increased clearly from acute to recovery phases (Figure 3), and both of these were among the most dynamic cell populations during severe disease, which is suggestive of important contributions to antiviral defense and immunopathology.

### Eosinophil Activation and Homing during Acute COVID-19

**Figure 4:**
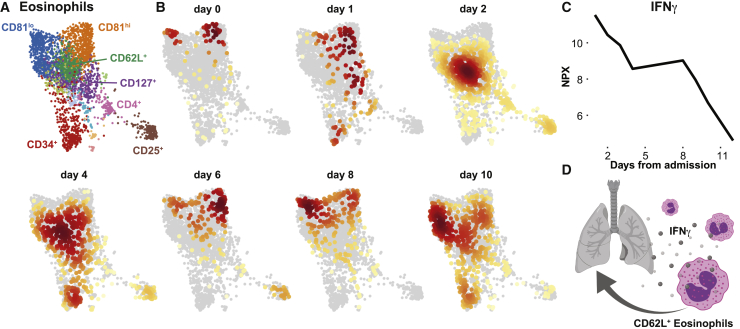
Eosinophil Changes from Admission to Recovery 2D representation generated by partition-based graph abstraction (PAGA) of eosinophils from patient COV-40 at 7 different time points from admission to recovery. (A and B) Louvain clusters are colored and annotated by key protein characteristics (A), and cell distributions at each individual time point indicate changes in immune cell states and composition over time (B). (C) Plasma IFN-γ levels as measured by Olink assay in plasma samples from patient COV-40. (D) IFN-γ-mediated upregulation of CD62L contributes to lung inflammation hyperinflammation.

Given the changes in eosinophil abundance described above, we decided to study eosinophils more carefully. There are reports of strong granulocyte-macrophage colony-stimulating factor (GM-CSF) responses in the lungs of COVID-19 patients,^14^ and GM-CSF is known to stimulate eosinophils, particularly in interstitial pneumonia and allergic inflammation.^15^ Taking advantage of the detailed longitudinal sample series, we used partition-based graph abstraction (PAGA),^16^ to reconstruct single-cell phenotypic changes in blood eosinophils during acute COVID-19 (Figure 4). Leiden clustering found 12 eosinophil subsets, and the main groups are annotated by defining features (Figure 4A). By splitting cells obtained from the different longitudinal samples, time-associated changes in eosinophil phenotypes were revealed, with a transient expansion of CD62L^+^ eosinophils from days 2–6 after admission (Figure 4B). CD62L upregulation on eosinophils has been reported to be induced by IFN-γ,^17^ one of the most elevated cytokines in severe COVID-19, and the IFN-γ levels show a slight increase right around the same time as the expansion of CD62L^+^ eosinophils (Figure 4C). This phenotype of eosinophils is reminiscent of lung-resident eosinophils, rather than induced inflammatory eosinophils in circulation, and such lung-homing cells have previously been reported to be important homeostatic regulators of inflammatory responses in the lung^18^ (Figure 4D). It is tempting to think that this transient expansion of CD62L^+^ eosinophils just before the development of severe lung hyperinflammation at ∼1 week after admission is related to this immunopathology of the lungs in COVID-19 patients. To this end, further investigation into this eosinophil-IFN-γ axis is required and may suggest novel therapies targeting this response to mitigate acute respiratory distress syndrome (ARDS) and lung inflammation.

### Adaptive Immune Cell Dynamics during Severe COVID-19

**Figure 5:**
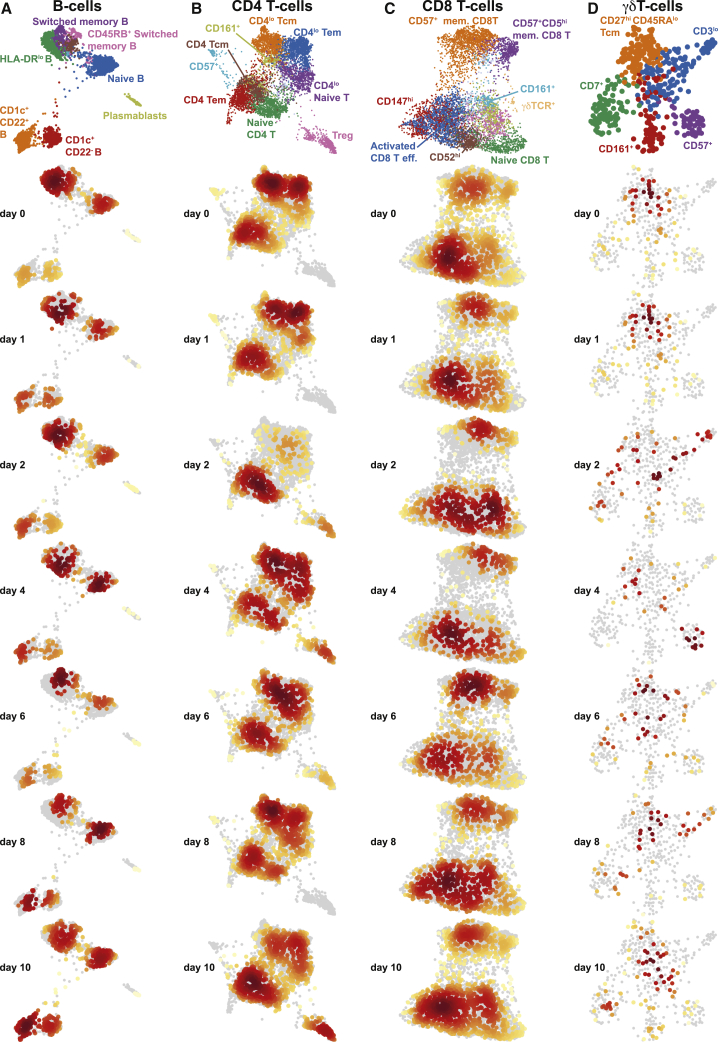
Adaptive Immune Cell Changes from Admission to Recovery 2D representation generated by PAGA B cells (A), CD4 T cells (B), CD8 T cells (C), and γδT cells from patient COV-40 at 7 different time points from admission to recovery. The Louvain clusters are colored (top) and annotated by key protein characteristics, and cell distributions at each individual time point indicate changes in immune cell states and composition over time.

Adaptive responses to SARS-CoV-2 are seen in most individuals, with one study reporting CD4^+^ T cell responses and CD8^+^ T cell responses in nearly all patients.^19^ Similarly, the majority of symptomatic patients seroconvert within a few days and most developed high-titer antibody responses^20^; however, one study has reported that a significant proportion of patients with COVID-19 do not develop neutralizing antibody responses.^21^ To investigate the dynamics of adaptive immune cell responses in our cohort, we used the same PAGA approach as described above. We find a clear plasmablast response early after admission (Figure 5A). The CD4^+^ T cell response was initially dominated by effector and central memory responses, followed by an increase in regulatory T cells (Tregs) ∼4 days after admission (Figure 5B). The CD4^+^ T cells were split into two effector cell populations based on CD4 expression level, possibly reflecting an activation-induced downregulation in a subset of CD4^+^ T cells (Figure 5B).^22^ The CD8^+^ T cell responses are dominated by activated cells expressing high CD38 and also a subset of effector cells upregulating the CD147 receptor from ∼1week onward (Figure 5C). Gamma-delta T cell receptor (TCR) T cells (γδT cells) and CD8^+^ T cells progressively upregulated the marker of terminal maturation CD57 from ∼1 week onward (Figures 5C and 5D). These results are largely in agreement with other recent reports^23^ and highlight the strong innate and adaptive immune activation during acute COVID-19.

### Cell-Cell Regulation Varies over Time during Severe COVID-19

**Figure 6:**
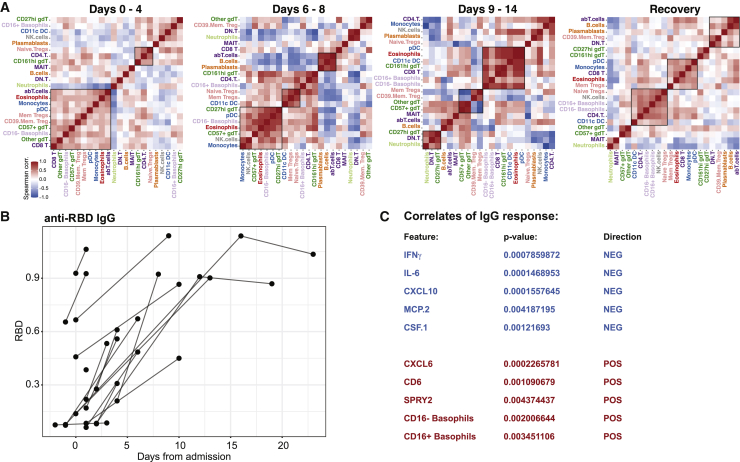
Cell-Cell Communications Network during Different Phases from Acute to Recovery of COVID-19 (A) Spearman correlation matrices from 35 patients, with samples collected at the indicated time intervals and ordered by top correlations. Co-regulated cell populations are highlighted by boxes. (B) Serum IgG antibodies against SARS-CoV-2 Spike protein receptor-binding domain (RBD) from 17 acute patients. RBD showed against days after admission. (C) Mixed-effects modeling (MEM) of plasma protein levels and immune cell population frequencies against anti-RBD IgG titers. The 5 most positively and negatively associated features in MEM are correlated with antibody responses when days from admission is taken into account as a fixed effect.

Immune responses are always concerted efforts made by multiple, specialized cell populations communicating via direct interactions and secreted cytokines and other mediators. By studying such cell-cell relationships, a better understanding of the systems-level response can be obtained. We generated cell-cell correlation matrices using longitudinal cell population frequencies and binned the samples into four phases, from acute disease to recovery phase (Figure 6A). We find that the first phase (days 0–4) is dominated by an inverse correlation between neutrophils and a number of myeloid and lymphoid cell types, as reflected in the elevated NLR, associated with severe disease^12^ (Figure 6A). The following phase (days 6–8) is characterized by a strong coordinated plasmablast, B cell, and αβT cell module, and this is inversely correlated with a strong Treg and CD11c^+^ DC module (Figure 6A). From day 9 onward, a change is apparent, with a shift toward a co-regulated module involving eosinophils, pDCs, CD11c^+^ DCs, and CD8^+^ T cells. This module is largely maintained in recovered patients, possibly reflecting a more normal cell-cell relationship (Figure 6A).

A prototype of a coordinated immune response to viruses is the appearance of virus-specific immunoglobulin G (IgG) antibodies, because such responses elicited by B cells require help from CD4^+^ T cells. Here, we investigated the seroconversion in this cohort and found a strong induction of IgG antibodies to the SARS-CoV-2 Spike protein (receptor-binding domain [RBD]) in the majority of patients (Figure 6B). This is in line with similar analyses in other COVID-19 patients.^20^^,^^24^ We were unable to test the neutralizing capacity of these antibodies at the time of the study, but another recent report has shown that a significant proportion of patients mount antibodies that lack such neutralizing capacity.^21^ To understand the immunological correlates of IgG responses to SARS-CoV-2, we devised a mixed-effects model, using both plasma protein levels and cell frequencies as predictors, taking days after admission into account as a fixed effect.^25^ It is important to note that this is not a simple correlation analysis since days from admission is taken into account as a fixed effect in the analysis. We found several features significantly associated with IgG responses, and in particular, strong proinflammatory cytokines IFN-γ and IL-6 and chemokines CXCL10 and MCP-2 (CCL8) are negatively associated with anti-CoV-2 IgG responses (Figure 6C). In contrast, the neutrophil-recruiting chemokine CXCL6 is positively associated with anti-CoV-2 IgG responses as was the fraction of circulating basophils (Figure 6C). It is known that basophils are able to bind antigens on their surface and potentiate humoral immune responses^26^; since basophils are depleted during acute and severe COVID-19 (Figure 3), our data collectively suggest that the degree of basophil depletion may influence the efficacy of IgG responses to SARS-CoV-2. It is believed that basophil-mediated enhancement of B cell responses occurs through the production of either IL-4 or IL-6, but levels of the latter were found to be inversely associated with antibody responses (Figure 6C), so it is more likely that another mechanism is responsible for the basophil enhancement of IgG responses in COVID-19. Collectively, these results indicate a coordinate adaptive immune response to SARS-CoV-2, enhanced by basophils and possibly suppressed by hyperinflammatory cytokine responses with high IL-6 levels during acute COVID-19.

### A Shared, Integrated Trajectory of Recovery across Patients

Since none of the patients in this cohort were treated with immunomodulatory agents, apart from one patient who received Oxiklorin treatment before ICU admission, and have recovered with supportive care alone, we reasoned that a deeper investigation into the immunological changes during recovery from severe COVID-19 would be informative about the underlying immune processes involved. Given the strong interactions among immune cells and proteins in the immune system, we applied an integrative analysis method to search for a multiomic trajectory of immune recovery. We used multiomics factor analysis (MOFA).^27^ This method allowed us to search for the latent factors (LFs) that best explain the variance across data types and use these to discern any possible relationship with the process of recovery from the disease.

**Figure 7:**
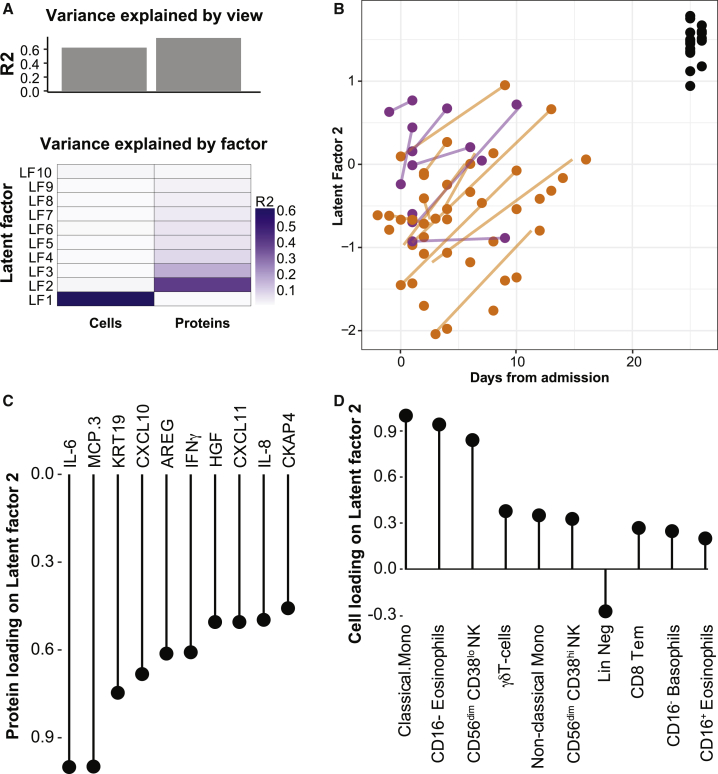
A Multiomics Immune Signature from Acute COVID-19 to Recovery Multiomics factor analysis (MOFA) is used to integrate 148 plasma protein levels and 63 immune cell frequencies across all 96 blood samples collected from 37 patients. (A) Fraction of total variance explained by type of measurement (view) and by latent factors (LFs) 1–10. (B) LF2 best represents the changes from acute to recovery over time and reveals a shared trajectory for most patients (non-ICU shown in purple and ICU shown in orange). (C) Lollipop plot shows plasma proteins explaining LF2. (D) Lollipop plot shows cell population frequencies explaining LF2.

We found 10 LFs that explained the variance in the combined dataset (Figure 7A), and of those, LF2 was associated with the transition from acute to recovery phases of the disease (Figure 7B). There were no clear differences among ICU or non-ICU ward patients, and the levels of LF2 were highest in the samples taken from recovered patients (Figure 7B). To understand the biology of immune recovery, we assessed the underlying features contributing to LF2. The plasma proteins changing the most decreased during recovery. The most prominent were IL-6, monocyte-chemotactic protein 3 MCP3, Keratin19 (KRT19), CXC chemokine ligand 10 (CXCL10), amphiregulin (AREG), and IFN-γ (Figure 7C). Conversely, the cells that changed the most during recovery were classical and non-classical monocytes, CD56^dim^ natural killer (NK) cells, eosinophils, and γδT cells, all increasing in relative proportions during recovery (Figure 7D). This shared, integrated trajectory reveals the markers most indicative of recovery in patients with severe COVID-19, and if verified in independent sets of patients, these features could be valuable biomarkers to monitor during disease progression to detect a switch from acute to recovery phases in severe COVID-19.

In this article, we have performed an in-depth, longitudinal analysis of the immune system in patients with severe COVID-19 during acute disease and up until spontaneous recovery. The natural course of this process is mapped and found to be similar among patients. We find changes in cell populations, such as CD62L-expressing eosinophils, triggered by IFN-γ and likely contributing to hyperinflammation and ARDS during acute disease. We show that basophils are depleted during acute disease but recuperate during recovery, and the levels of basophils are significantly correlated with the titers of IgG antibodies to SARS-CoV-2 produced by B cells. In contrast, high levels of IL-6 and IFN-γ are negatively associated with humoral responses. Finally, we uncover an immunological trajectory of disease recovery shared among patients. These results can be useful for the development of better immunomodulatory strategies to mitigate hyperinflammatory responses, optimize antiviral IgG responses, and monitor disease progression and recovery in patients with severe COVID-19.

## Discussion

A number of researchers are studying the immune response to SARS-CoV-2, and we are learning about viral evasion of IFN-I/III signals and prevention of the normal induction ISGs and the antiviral state.^4^^,^^5^ At the same time, the proinflammatory response is strong. The secretion of chemokines and proinflammatory cytokines leads to the influx of neutrophils and myeloid cells into the lung, with strong local inflammatory responses and immunopathology.^6^ Autopsy findings in patients who have succumbed to COVID-19 are characterized by perivascular T cell infiltration, microangiopathy, and widespread thrombosis in lung tissue.^28^ The induction of IL-6 during severe COVID-19 has led to trials of blocking antibodies to the IL-6 receptor with mixed results. This is inspired by cytokine release syndrome (CRS), seen in cancer immunotherapy, which is also often treated with IL-6-blocking agents. However, there are a number of differences between severe COVID-19 and CRS, such as lower IL-6 levels and death caused by respiratory failure and thrombosis, rather than from circulatory failure and status epilepticus, as seen in CRS.^29^ In this respect, the mechanisms of severe COVID-19 are incompletely understood, and better understanding is required for improved immunomodulatory therapies to be devised and immunopathology and mortality limited.

Human immune systems are highly variable,^30^ and most of this variation is explained by environmental exposures,^31^ particularly early in life.^32^ The role of genetic variation in immune variation in general and in COVID-19 in particular is under investigation.^33^ Systems-level analysis methods are useful in human immunology because they capture the many variable cell populations, proteins, and transcriptional programs involved in a complex immune response. Systems-level analyses also allow for the inference of relationships among such immune system components.^34^ With this study, we add to the rapidly growing literature by providing a longitudinal, systems-level perspective on the immune system changes from acute to recovery phases of severe COVID-19 disease. Longitudinal analyses are important because cross-sectional analyses carry the risk of capturing snapshots of patients at different stages of the immune response and thereby misinterpret differences as qualitatively different. The longitudinal sampling presented herein is a strength of this study. Another important aspect of this work is its use of whole blood, rather than peripheral blood mononuclear cells, allowing neutrophils and other granulocyte populations to be included in the analysis and also reduce the technical sources of variation caused by cell preparation and freezing.^35^ By using this more holistic and longitudinal approach to analyze the immune response during severe COVID-19, we find previously unappreciated roles of eosinophils in the acute response. These cells play important roles in other respiratory infections,^36^ but they have not been implicated much in COVID-19. The population of eosinophils that expand a few days after admission to the hospital were characterized by high CD62L expression, a previously reported marker of lung eosinophils,^18^ and it is possible that such IFN-γ-mediated upregulation of CD62L on eosinophils leads to the influx of these cells into the lung tissue. The development of ARDS and clinical deterioration is typically seen after ∼1 week in severe patients, and it is possible that this IFN-γ-eosinophil axis contributes to this, but a causal role is beyond the scope of our study. The finding that basophil levels are positively associated with humoral responses to SARS-CoV-2 is intriguing and in line with previous studies in other viral infections.^26^ Further investigation will be required to understand the mechanisms involved, but it likely would not involve the production of IL-6 by basophils, given that plasma levels of this cytokine were inversely associated with anti-RBD IgG titers. Another possible mechanism involves IL-4 production by basophils, known to potentiate B cell responses to infection in other settings.^37^ It is worth noting that time after admission is taken into account in the mixed-effects model, and the reported associations are the significant ones after time from admission is taken into account.

There has been a lot of concern around antibody responses to SARS-CoV-2, and although nearly all of the patients with severe disease do produce antibodies in rather high titers,^19^^,^^24^ the neutralizing capability of such antibodies are variable.^21^ One hypothesis brought forward as a possible explanation of the severe disease occurring often after 1 week or so of stable disease is antibody-dependent enhancement (ADE).^38^^,^^39^ This occurs when non-neutralizing antibodies bind a virus and via Fc receptors bring viruses into new cell types, not expressing the receptor required for viral entry—in this case, ACE2. Such responses are well known for dengue virus infection and could induce hyperinflammatory responses also in COVID-19. We have found that a significant proportion of CD4^+^ T cells in some patients showed CD4 downregulation as a sign of possible cell activation, but such downregulation can also occur if T cells are directly infected.^40^ CD4^+^ T cells do not express ACE2,^41^ but they could express Fc receptors and thus be subject to viral infection and replication via ADE. This is speculative at this time, but as more data surface on determinants of neutralizing antibody responses, the theory of ADE as a cause of severe COVID-19 will be testable and have important implications for vaccine development.^38^ The influence of basophils in modulating humoral responses to SARS-CoV-2 uncovered herein should also be taken into account, as basophils are depleted during acute disease and the severity of such depletion may be an important determinant of the antibody response to the virus.

### Limitations of Study

This study has several limitations. We performed longitudinal systems-level immunomonitoring of acute and recovered COVID-19 patients, but due to logistical limitations in the overwhelmed hospital wards, we were unable to collect longitudinal samples for recovered patients and were also limited in the number of acute COVID-19 disease patients we could enroll. We were unable to include a healthy cohort, and we were not powered to robustly compare patients in ICU versus non-ICU with respect to their immunological trajectories.

## STAR★Methods

### Key Resources Table

**Table:** 

REAGENT or RESOURCE	SOURCE	IDENTIFIER
**Mass cytometry – Broad extended panel**		

Anti-human CD1c (L161), Purified	Biolegend	Cat# 331502; RRID: AB_1088995
Anti-human CD3e (UCHT1), Purified	Biolegend	Cat# 317302; RRID: AB_571927
Anti-human CD4 (RPA-T4), Purified	Biolegend	Cat# 300502; RRID: AB_314070
Anti-human CD5 (UCHT2), Purified	Biolegend	Cat# 300602; RRID: AB_314088
Anti-human CD7 (CD7-6B7), Purified	Biolegend	Cat# 343102; RRID: AB_1659214
Anti-human CD8 (SK1), Purified	Biolegend	Cat# 344702; RRID: AB_1877104
Anti-human CD9 (SN4 C3-3A2), Purified	eBiosciences	Cat# 14-0098-82; RRID: AB_657777
Anti-human CD11c (Bu15), Purified	Biolegend	Cat# 337202; RRID: AB_1236381
Anti-human CD14 (M5E2), Purified	Biolegend	Cat# 301802; RRID: AB_314184
Anti-human CD16 (3G8), Bi-209	Fluidigm	Cat# 3209002B, RRID: AB_2756431
Anti-human CD20 (2H7), Purified	Biolegend	Cat# 302302; RRID: AB_314250
Anti-human CD22 (HIB22), Purified	Biolegend	Cat# 302502; RRID: AB_314264
Anti-human CD24 (ML5), Purified	Biolegend	Cat# 311102; RRID: AB_314851
Anti-human CD25 (2A3), Sm-149	Fluidigm	Cat# 3149010B, RRID: AB_2756416
Anti-human CD26 (BA5b), Purified	Biolegend	Cat# 302702; RRID: AB_314286
Anti-human CD27 (L128), Er-167	Fluidigm	Cat# 3167006B; RRID: N/A
Anti-human CD28 (CD28.2), Purified	Biolegend	Cat# 302902; RRID: AB_314304
Anti-human CD29 (TS2/16), Purified	Biolegend	Cat# 303002; RRID: AB_314318
Anti-human CD33 (WM53), Purified	Biolegend	Cat# 303402; RRID: AB_314346
Anti-human CD34 (581), Purified	Biolegend	Cat# 343502; RRID: AB_1731898
Anti-human CD38 (HIT2), Purified	Biolegend	Cat# 303502; RRID: AB_314354
Anti-human CD39 (A1), Purified	Biolegend	Cat# 328202; RRID: AB_940438
Anti-human CD43 (84-3C1), Purified	eBiosciences	Cat# 14-0439-82; RRID: AB_763493
Anti-human CD45 (HI30), Y-89	Fluidigm	Cat# 3089003B; RRID: AB_2661851
Anti-human CD45RA (HI100), Tm-169	Fluidigm	Cat# 3169008B; RRID: N/A
Anti-human CD45RB (MEM-55), Purified	Biolegend	Cat# 310202; RRID: AB_314805
Anti-human CD49d (9F10), Pr-141	Fluidigm	Cat# 3141004B; RRID: N/A
Anti-human CD52 (HI186), Purified	Biolegend	Cat# 316002; RRID: AB_389275
Anti-human CD55 (JS11), Purified	Biolegend	Cat# 311302; RRID: AB_314859
Anti-human CD56 (NCAM16.2), Purified	BD	Cat# 559043; RRID: AB_397180
Anti-human CD57 (HCD57), Purified	Biolegend	Cat# 322302; RRID: AB_535988
Anti-human CD62L (DREG-56), Purified	Biolegend	Cat# 304802; RRID: AB_314462
Anti-human CD64 (10.1), Purified	Biolegend	Cat# 305002, RRID: AB_314486
Anti-human CD81 (5A6), Purified	Biolegend	Cat# 349502; RRID: AB_10643417
Anti-human CD85j (GHI/75), Purified	Biolegend	Cat# 333702; RRID: AB_1089089
Anti-human CD95 (DX2), Purified	Biolegend	Cat# 305602; RRID: AB_314576
Anti-human CD99 (HCD99), Purified	Biolegend	Cat# 318002; RRID: AB_604112
Anti-human CD123 (6H6), Purified	Biolegend	Cat# 306002; RRID: AB_314576
Anti-human CD127 (A019D5), Ho-165	Fluidigm	Cat# 3165008B; RRID: N/A
Anti-human CD137 (4B4-1), Purified	Biolegend	Cat# 309802; RRID: AB_314781
Anti-human CD141 (M80), Purified	Biolegend	Cat# 344102; RRID: AB_2201808
Anti-human CD147 (HIM6), Purified	Biolegend	Cat# 306202; RRID: AB_314586
Anti-human CD161 (HP-3G10), Purified	Biolegend	Cat# 339902; RRID: AB_2661837
Anti-human CX3CR1 (8E10.D9), Purified	Biolegend	Cat# 824001; RRID: AB_2564876
Anti-human HLA-DR (L243), Purified	Biolegend	Cat# 307602; RRID: AB_314680
Anti-human IgD (IA6-2), Purified	Biolegend	Cat# 348202; RRID: AB_10550095
Anti-human Siglec-8 (837535), Purified	R&D Systems	Cat# MAB7975; RRID: N/A
Anti-human TCRgd (5A6.E9), Purified	Fischer Scientific	Cat# TCR1061; RRID: AB_223500

**Biological Samples**		

Patients with COVID-19 from Helsinki	Hospital District of Helsinki and Uusimaa, Finland	N/A

**Chemicals, Peptides, and Recombinant Proteins**		

Bovine Serum Albumin	Sigma-Aldrich	Cat# A3059; RRID: N/A
Cell-ID Cisplatin Pt194	Fluidigm	Cat# 201194; RRID: N/A
Cell-ID Cisplatin Pt198	Fluidigm	Cat# 201198; RRID: N/A
Cisplatin Pt195	BuyIsotope	Customized
Cisplatin Pt196	BuyIsotope	Customized
Cell-ID Intercalator-Ir	Fluidigm	Cat# 201192B; RRID: N/A
Cell-ID 20-Plex Pd Barcoding Kit	Fluidigm	Cat# 201060; RRID: N/A
DMSO	Sigma-Aldrich	Cat# D8418; RRID: N/A
EDTA	Rockland	Cat# MB-014; RRID: N/A
EQ Four Element Calibration Beads	Fluidigm	Cat# 201078; RRID: N/A
FBS	Sigma-Aldrich	Cat# 12103C; RRID: N/A
Fc Receptor (FcR) blocking buffer	Cytodelics	Customized
Maxpar Cell Acquisition Solution (CAS)	Fluidigm	Cat# 201240; RRID; N/A
Maxpar MCP9 Antibody Labeling Kit −110Cd	Fluidigm	Cat# 201110A; RRID: N/A
Maxpar MCP9 Antibody Labeling Kit −111Cd	Fluidigm	Cat# 201111A; RRID: N/A
Maxpar MCP9 Antibody Labeling Kit −112Cd	Fluidigm	Cat# 201112A; RRID: N/A
Maxpar MCP9 Antibody Labeling Kit −113Cd	Fluidigm	Cat# 201113A; RRID: N/A
Maxpar MCP9 Antibody Labeling Kit −114Cd	Fluidigm	Cat# 201114A; RRID: N/A
Maxpar Water	Fluidigm	Cat# 201069; RRID; N/A
Maxpar X8 Multimetal Labeling Kit (40 rxn)	Fluidigm	Cat# 201300; RRID; N/A
Metal isotopes as chloride salts (In-115, Gd-155, Gd-157, Dy-161, Dy-163, Yb-173)	Trace Sciences International	Customized
Paraformaldehyde	VWR	Cat# 16005; RRID: N/A
Penicillin-streptomycin	Sigma-Aldrich	Cat# P4333; RRID: N/A
Protein Stabilizer PBS	Candor Bioscience	Cat# 131125, RRID: N/A
PBS 1X	Rockland	Cat# MB-008; RRID: N/A
RPMI 1640 medium	Sigma-Aldrich	Cat# R848; RRID: N/A
Sodium Azide	Sigma-Aldrich	Cat# 71289; RRID: N/A
Whole blood (human) processing kit	Cytodelics	Cat# hC001-500; RRID: N/A

**Critical Commercial Assays**		

Immune Response panel	Olink AB	N/A
Inflammation panel	Olink AB	N/A

**Other**		

BenchBot robot	Agilent technologies	Customized
Bravo liquid handling platform	Agilent technologies	Customized
CyTOF 2 upgraded Helios mass cytometer	Fluidigm	N/A
EL406 Washer Dispenser	BioTek	Customized
Helios mass cytometer	Fluidigm	N/A
pluriStrainer Mini, 40 μm	pluriSelect	Cat# 43-10040-70; RRID: N/A
Polypropylene tubes	Sarstedt	Cat# 55526; RRID: N/A
TC20 automated cell counter	BioRad	N/A
Vspin microplate centrifuge	Agilent technologies	Customized

**Deposited Data**		

FCS files, Mass cytometry	This paper	https://brodinlab.com/data-repository/
Protein expression data	This paper	https://brodinlab.com/data-repository/
IgG data	This paper	Table S1

**Software and Algorithms**		

CyTOF software (v. 6.5.358)	N/A	https://www.fluidigm.com/
Mass Cytometry Normalizer	Finck et al., 2013^47^	https://github.com/nolanlab/bead-normalization/releases
R 3.6.0	R Core Team, 2019	https://www.r-project.org/
Python 3.7.0		https://www.python.org/
Mass Cytometry Debarcoder	Zunder et al., 2015^48^	https://github.com/nolanlab/single-cell-debarcoder
CellGrid v0.5.5	Chen et al.^13^	https://github.com/Brodinlab/cellgrid
MOFA	Argelaguet et al.^27^	https://github.com/bioFAM/MOFA
PAGA	Wolf et al.^16^	https://github.com/theislab/paga

### Resource Availability

#### Lead Contact

Further information and requests for resources and reagents should be directed to and will be fulfilled by the Lead Contact, Petter brodin (petter.brodin@ki.se).

#### Materials Availability

This study did not generate new unique reagents.

#### Data and Code Availability

The raw mass cytometry data and the Olink protein data generated during this study are available for download on our lab webpage (https://brodinlab.com/data-repository/). The IgG data is presented in Table S1. Code used in the analyses is available here: https://github.com/rodriluc/SARS-CoV2_study.

### Experimental Model and Subject Details

#### Human Subjects

##### Inpatients

We included symptomatic patients with positive SARS-CoV-2 PCR test admitted to Helsinki University Hospital, Helsinki, Finland. Patients were recruited within five days after hospitalization. We excluded patients who had been considered by the attending clinician not to benefit from intensive care. The clinical decisions were based on comorbidities and general frailty, not the severity of the COVID-19 disease. We recruited 17 inpatients (9 females, 8 males) aged between 40 - 77 years. The duration of hospitalization ranged from 5 to 38 days. Of these, 10 were admitted to the ICU, and remained in intensive care for 1 - 27 days. Three patients required mechanical ventilation for 3, 13 and 19 days, respectively.

#### Recovery phase patients

In addition to patients recruited during the acute phase of illness, we recruited a separate cohort of recovered patients based on positive PCR (n = 20). Of this recovered cohort, there were 18 mild COVID-19 patients and 2 hospitalized COVID-19 patients. These 20 subjects (age range 28 - 68 years; 11 females, 9 males) were included during convalescence 3-4 weeks after COVID-19 diagnosis and SARS-CoV-2 detection. These patients were identified from medical and laboratory records, contacted by phone and invited to donate a blood sample. Characteristics of all patients described in Table S2.

This non-interventional, observational study was approved by the Ethics Committee of the Hospital District of Helsinki and Uusimaa (HUS/853/2020) and conducted in accordance with the Declaration of Helsinki. Written informed consent was obtained from all participants.

### Method Details

#### Immunophenotyping by Mass Cytometry

Blood samples drawn from patients with COVID-19 were mixed with a whole blood stabilizer^35^(Cytodelics AB, Sweden) either immediately or within 1-3 hr post blood draw and cryopreserved as per the manufacturer’s recommendations. Samples were then thawed, and cells were fixed/RBCs lysed using WASH # 1 and WASH # 2 buffers (Whole blood processing kit; Cytodelics AB, Sweden) as per the manufacturer’s recommendations. This was performed a few days prior to barcoding and staining of cells. Post fix/lysis of cells, ∼1-2x10^6^ cells/sample were plated onto a 96 well round bottom plate using standard cryoprotective solution (10% DMSO and 90% FBS) and cryopreserved at −80°C.

At the time of experimentation, cells were thawed at 37°C using RPMI medium supplemented with 10% fetal bovine serum (FBS), 1% penicillin-streptomycin and benzonase (Sigma-Aldrich, Sweden). Briefly, cells were barcoded using automated liquid handling robotic system (Agilent technologies)^42^ using the Cell-ID 20-plex Barcoding kit (Fluidigm Inc.) as per the manufacturer’s recommendations. Samples were pooled batch-wise by keeping together the longitudinal samples from each patient in the same batch. Cells were then washed, FcR blocked using blocking buffer (in-house developed recipe) for 12 min at room temperature, following which cells were incubated for another 30 min at 4°C after addition of a cocktail of metal conjugated antibodies targeting the surface antigens. Cells were washed twice with CyFACS buffer (PBS with 0.1% BSA, 0.05% sodium azide and 2mM EDTA) and fixed overnight using 2% formaldehyde made in PBS (VWR, Sweden). The broad extended panel of antibodies used are listed in Key Resources Table. For acquisition by CyTOF, cells were stained with DNA intercalator (0.125 μM Iridium-191/193 or MaxPar® Intercalator-Ir, Fluidigm) in 2% formaldehyde made in PBS for 20 min at room temperature. Cells were washed once with CyFACS buffer, PBS and Milli-Q water, and twice with Cell acquisition solution (CAS) (Fluidigm). Cells were mixed with 0.1X Norm Beads (EQ™ Four Element Calibration Beads, Fluidigm) filtered through a 35μm nylon mesh and diluted to 1000,000 cells/ml. Cells were acquired using Helios mass cytometer at a rate of 300-500 cells/s using PSI system, CyTOF software version 6.5.358 with noise reduction, a lower convolution threshold of 400, event length limits of 10-150 pushes, a sigma value of 3, and flow rate of 0.030 ml/min.

#### Antibodies and reagents

Purified antibodies for mass cytometry were obtained in carrier/protein-free buffer and then coupled to lanthanide metals using the MaxPar antibody conjugation kit (Fluidigm Inc.) as per the manufacturer’s recommendations. Following the protein concentration determination by measurement of absorbance at 280 nm on a nanodrop, the metal-labeled antibodies were diluted in Candor PBS Antibody Stabilization solution (Candor Bioscience, Germany) for long-term storage at 4°C. Antibodies used are listed in Key Resources Table.

#### Plasma protein profiling

Serum or plasma samples collected from patients with COVID-19 (by spinning blood at 2000 g for 10min at 80 C for plasma collection or by collecting serum from those blood samples from which PBMCs were isolated using gradient centrifugation for future use and not intended for this study) were analyzed using a multiplex proximity extension assay (OLINK Bioscience, Uppsala, Sweden). Each kit provides a microtiter plate for measuring 92 protein biomarkers. Two panels, the Olink Immune Response and Inflammation panels. Each well contains 96 pairs of DNA-labeled antibody probes. Samples were incubated in the presence of proximity antibody pairs tagged with DNA reporter molecules. When the antibody pair bounds to their corresponding antigens, the corresponding DNA tails form an amplicon by proximity extension, which can be quantified by high-throughput real-time PCR.

#### Detection of anti-SARS-CoV-2 antibody response

Antibodies against SARS-CoV-2 were measured using indirect immunofluorescence assay (IFA) and enzyme-linked immunosorbent assay (ELISA) using SARS-CoV-2 receptor-binding domain (RBD) as the antigen. The IFA was conducted as described.^43^ The RBD ELISA was done following a recently published^24^^,^^44^ protocol. The RBD antigen was produced by transient transfection of RBD plasmid to Vero E6 cells and the produced protein was purified following an established protocol.^44^ The raw data is available in Table S2.

### Quantification and Statistical Analysis

#### Mass Cytometry Preprocessing and Gating

All FCS-files unrandomized using the CyTOF software (version 6.0.626) were transferred without any additional preprocessing.

#### Automated Cell Classification

Grid is an in-house supervised algorithm based on the selection of all relevant FCS files and relevant phenotypic markers for clustering in order to manually gate for sub-populations to use as a reference.^13^ The reference is then used to train a classifier algorithm to categorize similar cells quickly and accurately. The following populations were gated: B cells (CD1c- naive B cells, CD1c- switched B cells, CD1c+ naive B cells, CD1c+ switched B cells, CD27 CD43 B cells, unswitched memory B cells, transitional B cells and plasmablasts), eosinophils (CD16- eosinophils and CD16+ eosinophils), monocytes (classical monocytes, intermediary monocytes, myeloid CD1c DC, non-classical monocytes, and other myeloid cells), natural killer (NK) cells (CD4 CD56+ T cells, CD56^bright^ NK, CD56^dim^ CD16 NK, CD56^dim^ CD38^low^ NK, CD56^dim^ CD38^high^ NK, CD8 CD56+ T cells, and other NK cells), neutrophils (classic neutrophils), abT-cells/CD4T (CD127 memory CD4T, CD24 CD16 naive CD4T, CD24 CD16 memory CD4T, CD39 memory Tregs, CD57 memory CD4T, memory CD4T, memory Tregs, naive CD4T, and naive Tregs), abT-cells/CD8T (activated memory CD8T, CD16+ naive CD8T, CD16+ memory CD8T, CD57+ memory CD8T, CD62L CD127 CD8 TCM, CD7^high^ memory CD8T, CD8 TEM, DP T cells, naive CD8T, and other memory CD8T cells), abT-cells/DN T cells, abT-cells/MAIT (CD16- MAIT and CD16+ MAIT), gdT (CD161^high^ gdT, CD27^high^ gdT, and CD57+ gdT), CD11c DC, CD16- basophils, CD16+ basophils, other lineage-negative cells, and pDC. These sub-populations were identified by phenotypic markers from the parameter selection.

#### Multiomics Factor Analysis, MOFA v1

Multiomics Factor Analysis (MOFA) was used to discover principal sources of variation in multi-omics datasets.^27^ MOFA uses a set of data matrices as input formatted with features as columns and samples as rows, plasma protein expression and cell abundance datasets were used to build the *MOFAobject* with *MultiAssayExperiment*. The fitting step includes training the model with the multi-omics data in order to be able to disentangle the heterogeneity into a small number of latent factors. The *MOFAobject* was trained in R through the *reticulate* package with 10 factors and a variance threshold of 0.01%. Both omics datasets were processed individually to remove any features resulting in zero or low variance before fitting the model. Convergence of the model was assessed using the change in ELBO (deltaELBO) to verify it fit the convergence threshold which is considered to be between 1 and 10. Multiple models were run under different initializations to validate that factors were consistent across trials for model selection. The fitted MOFA model could then be interrogated in R for downstream analysis to characterize these factors as technical or biological sources of variation.

#### Partition-based graph abstraction of single-cell data

The CyTOF data were first preprocessed with arcsin h and scaled to unit variance and then partitioned into different subpopulations according to our in-house supervised learning algorithm. For each subpopulation, the phenotypic changes over different time points are inferred with a trajectory inference method called PAGA.^16^ In brief, PCA was first applied to reduce the number of features to 20, and then an undirected kNN-like graph was constructed using the approximate nearest neighbor search within UMAP, while each node represents a single cell and each edge represents a neighborhood relationship. After the construction of graph, the highly connected clusters were detected with Leiden method.^45^ Afterward, the clusters defined by Leiden were used by PAGA to infer a trajectory map. In the trajectory map, Leiden clusters are considered as connected if their number of inter-connections is larger than a fraction of the number of inter-connections expected under random assignment, and the threshold fraction is determined by a statistical model. Finally, the PAGA graph was taken as the initial position of a manifold learning method ForceAtlas2 (FA)^46^ and produced topology-preserving single-cell embeddings for visualization.

#### Mixed effects modeling

A partially Bayesian method was applied with *blme* package on both datasets (plasma protein expression and cell abundance) to produce maximum *a posteriori* (MAP) estimates.^25^ This provided the ability to nest the variables, and account for days from admission as well as RBD levels as fixed effects. Wald p values of covariates were extracted from models to assess significance.

## Supplemental Information

Document S1. Figures S1 and S2 and Tables S1 and S2

Document S2. Article plus Supplemental Information
