# Supplementary material for: Systems-Level Immunomonitoring from Acute to Recovery Phase of Severe COVID-19
Source: Cell Rep Med. 2020 Aug 5;1(5):100078. doi: 10.1016/j.xcrm.2020.100078 (PMC7405891; doi:10.1016/j.xcrm.2020.100078)
Supplement: Document S1. Figures S1 and S2 and Tables S1 and S2 [file mmc1.pdf]

Cell Reports Medicine, Volume *1*

Supplemental Information

Systems-Level Immunomonitoring from Acute to Recovery Phase of Severe COVID-19

Lucie Rodriguez, Pirkka T. Pekkarinen, Tadepally Lakshmikanth, Ziyang Tan, Camila Rosat Consiglio, Christian Pou, Yang Chen, Constantin Habimana Mugabo, Ngoc Anh Nguyen, Kirsten Nowlan, Tomas Strandin, Lev Levanov, Jaromir Mikes, Jun Wang, Anu Kantele, Jussi Hepojoki, Olli Vapalahti, Santtu Heinonen, Eliisa Kekäläinen, and Petter Brodin

Suppl. Figure 1. Immune cell proportions in COVID-19 of ICU and recovered individuals, Related to Figure 3.

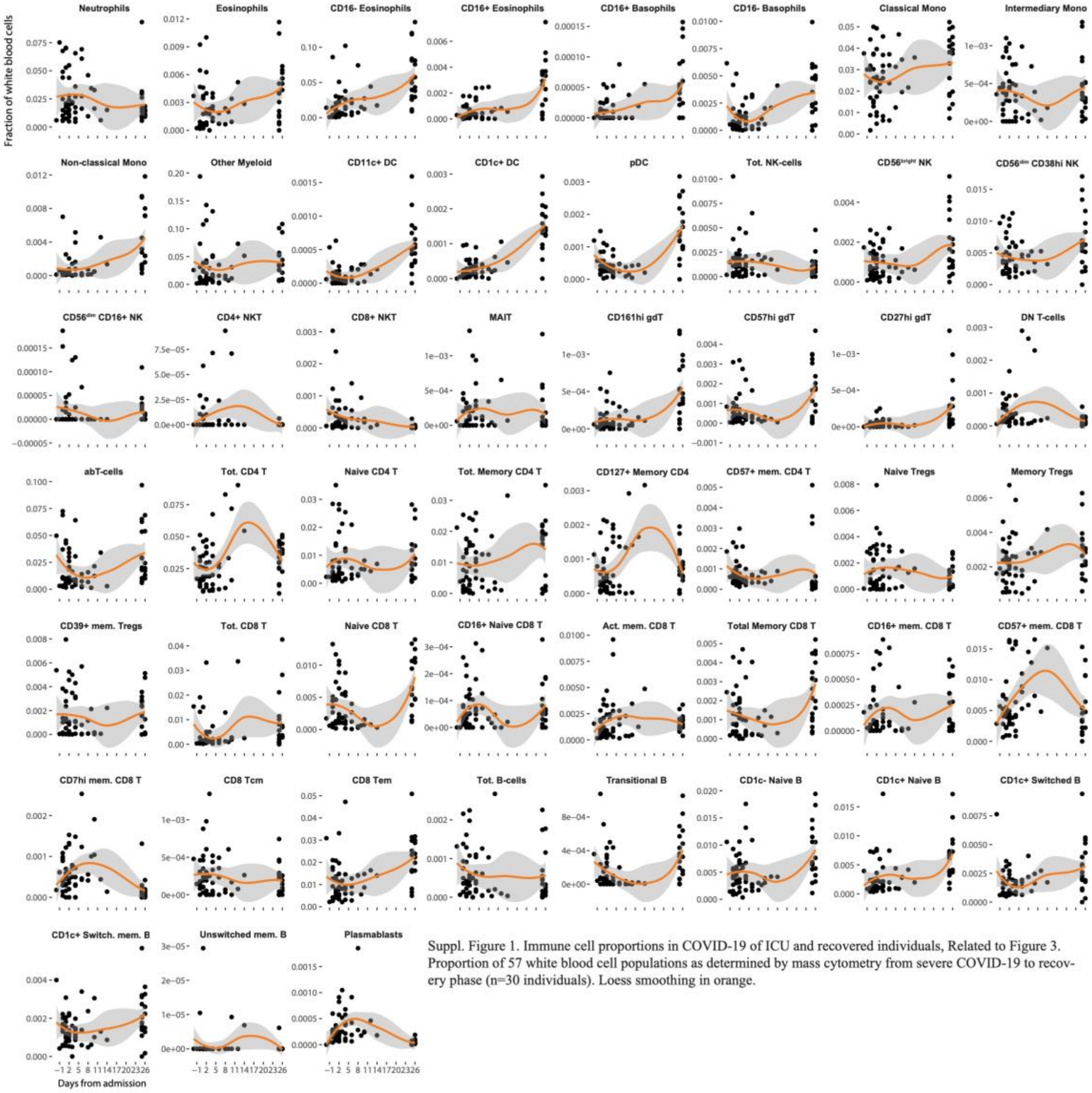

Suppl. Figure 1. Immune cell proportions in COVID-19 of ICU and recovered individuals, Related to Figure 3. Proportion of 57 white blood cell populations as determined by mass cytometry from severe COVID-19 to recovery phase (n=30 individuals). Loess smoothing in orange.

Suppl. Figure 2. Immune cell proportions in COVID-19 of Non-ICU and recovered individuals, Related to Figure 3.

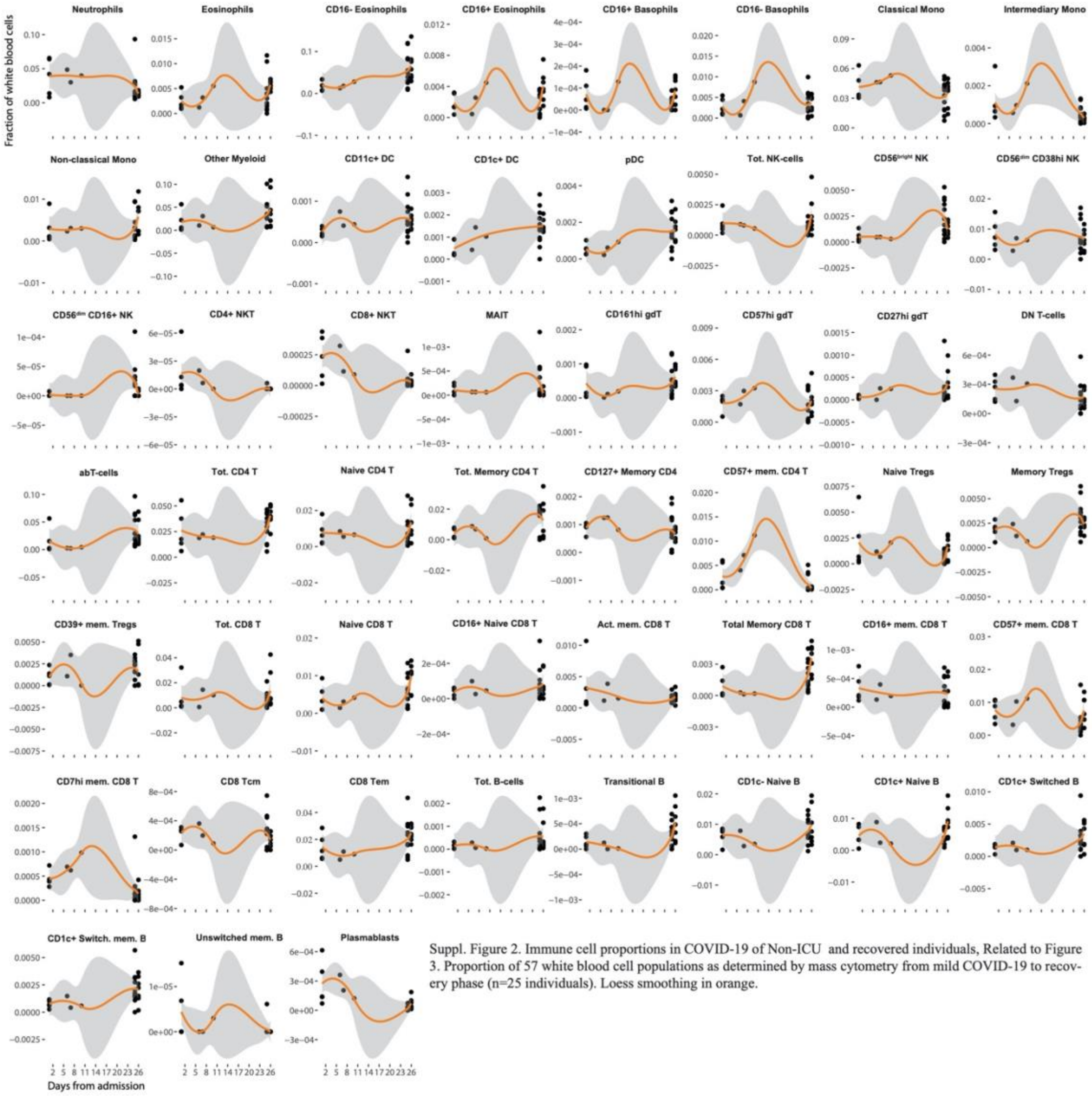

Suppl. Figure 2. Immune cell proportions in COVID-19 of Non-ICU and recovered individuals, Related to Figure 3. Proportion of 57 white blood cell populations as determined by mass cytometry from mild COVID-19 to recovery phase (n=25 individuals). Loess smoothing in orange.

Suppl. Table 1. IgG data, Related to STAR Methods.

Sample ID	Subject ID	IFA (inf. cells)	old-RBD	VE6-RBD
CoV-24, serum, t1	COV-24	320	0.138	0.064
CoV-24, heparin, t2	COV-24	2570	0.902	0.2025
CoV-25, serum, t1	COV-25	320	0.219	0.0795
CoV-25, EDTA, t2	COV-25	1280	0.533	0.118
CoV-26, serum, t1	COV-26	2560	0.458	0.1015
CoV-26, heparin, t2	COV-26	2570	0.866	0.163
CoV-27, serum, t1	COV-27	640	0.666	0.1415
CoV-27, heparin, t2	COV-27	1280	1.139	0.218
CoV-28, serum, t1	COV-28	640	0.385	0.1
CoV-29, serum, t1	COV-29	160	0.21	0.085
CoV-29, heparin, t2	COV-29	1280	0.923	0.1865
CoV-30, serum, t1	COV-30	1280	0.928	0.1895
CoV-30, serum, t2	COV-30	2570	1.063	0.215
CoV-31, serum, t1	COV-31	320	0.654	0.135
CoV-31, serum, t2	COV-31	640	0.926	0.185
CoV-32, serum, t1	COV-32	80	0.084	0.0645
CoV-32, heparin, t2	COV-32	1280	0.45	0.1035
CoV-33, serum, t1	COV-33	80	0.17	0.0685
CoV-33, heparin, t2	COV-33	640	0.559	0.112
CoV-34, EDTA, t1	COV-34	10	0.077	0.0625
CoV-34, EDTA, t2	COV-34	2560	1.138	0.267
CoV-34, EDTA, t3	COV-34	2560	1.035	0.237
CoV-35, serum, t1	COV-35	10	0.073	0.06
CoV-35, heparin, t2	COV-35	2560	0.672	0.141
CoV-36, serum, t1	COV-36	10	0.061	0.057
CoV-36, serum, t2	COV-36	640	0.485	0.117
CoV-37, serum, t1	COV-37	10	0.075	0.0615
CoV-37, heparin, t2	COV-37	80	0.086	0.063
CoV-39, serum, t1	COV-39	640	0.277	0.081
CoV-39, heparin, t2	COV-39	640	0.61	0.123
CoV-40, serum, t1	COV-40	1280	0.084	0.065
CoV-40, heparin, t2	COV-40	2570	0.909	0.2445
CoV-40, EDTA, t3	COV-40	2560	0.869	0.216
CoV-41, serum, t1	COV-41	320	0.081	0.0615

CoV-41, heparin, t2	COV-41	640	0.308	0.0985

Suppl. Table 2. Patient characteristics, Related to STAR Methods.

Subject ID	Age	Sex	ICU status	Acute (A) or Recovery (R)	Treatments/Conditions
COV-3	35	M		R	
COV-4	54	F		R	
COV-5	56	F		R	
COV-7	34	M		R	
COV-8	50	F		R	
COV-9	59	F		R	
COV-10	57	M		R	
COV-11	56	F		R	
COV-13	61	F		R	
COV-14	28	F		R	
COV-15	32	F		R	
COV-16	33	M		R	
COV-17	35	F		R	
COV-18	45	F		R	
COV-19	46	M		R	
COV-20	52	M		R	
COV-21	34	M		R	
COV-22	40	M		R	
COV-23	32	F		R	
COV-24	53	F	ICU	A	
COV-25	40	M	Non-ICU	A	
COV-26	77	M	ICU	A	
COV-27	59	F	ICU	A	
COV-28	52	M	Non-ICU	A	
COV-29	58	F	ICU	A	
COV-30	64	F	Non-ICU	A	
COV-31	57	F	Non-ICU	A	
COV-32	65	F	Non-ICU	A	
COV-33	55	M	Non-ICU	A	
COV-34	63	M	ICU	A	CA/CPR/epinephrine
COV-35	50	M	ICU	A	
COV-36	61	F	Non-ICU	A	
COV-37	62	M	ICU	A	
COV-39	46	F	ICU	A	Lactating
COV-40	69	M	ICU	A	
COV-41	55	F	ICU	A	Prior Oxychlorin treatment
COV-43	66	F		R	
